# Spousal abuse and its determinants

**DOI:** 10.1192/j.eurpsy.2021.1608

**Published:** 2021-08-13

**Authors:** S. Kolsi, S. Hentati, I. Baati, J. Masmoudi

**Affiliations:** Psychiatry A, CHU Hédi Chaker Sfax, Sfax, Tunisia

## Abstract

**Introduction:**

Spousal abuse (SA)against women, by its frequency and its consequences on the health of the victims, is a public health issue. For this reason, the role of the physician is essential not only in the care of victims but also in the study of the determinants of(SA).

**Objectives:**

To study the profile of women who have experienced(SA), their spouses and to evaluate the factors associated with spousal violence.

**Methods:**

Analytical and descriptive cross-sectional study conducted among married patients who consulted the National Health Fund of Sfax(CNSS) during the months of October and November 2019.The sociodemographic and clinical characteristics of the victims and their spouses were collected using a pre-established form.

**Results:**

57.3% of the population was affected by(SA). The mean age of female victims was 48.35 years(SD=9.82). 66.7% of women had a primary school level and 69% had a median socioeconomic level. The majority (60.3%) were housewives.78.18% had a somatic history. The average age of spouses was 53.82(SD=10.87).73% had an elementary school education and 49% were workers.The spouse’s somatic history was found in 63.5% and psychiatric history in 11.11%. 39.68% of spouses had addictive behaviours. Factors correlated with (SA) were: low education levels of the wife (p=0.016) and husband (p=0.0057), history of childhood abuse of the victim (p<0.0001), addictive behaviours of the husband (p=0.008).

**Conclusions:**

It seems that the evaluation of the characteristics of women victims of (SA) and their spouses, as well as the identification of factors associated with (SA), are essential in order to cope with this scourge and avoid its repercussions.

**Keyword:**

spousal abuse-victims-determinants-profile

